# Standard of Entrance Raised

**Published:** 1898-05

**Authors:** 


### STANDARD OF ENTRANCE RAISED.

There has been an effort for some time to raise the standard of entrance in dental colleges throughout the United States, and it is generally conceded that this will be required of all in the near future, but it is at the same time recognized that the colleges, as a whole, are not prepared to accede to the demands of some of the more favorably situated institutions.

The faculty of the Department of Dentistry of the University of Pennsylvania has recently adopted the following as its standard for the future:

"Candidates for admission will be required to present evidence of preliminary education as follows: For the session of 1898–99, a certificate of High School entrance; for the session of 1899–1900, a certificate of two years' High School attendance; for the session of 1900–1, a diploma of an approved High School having a three years' course, or a certificate showing three years' attendance at a High School having a four years' course, or certificates from other schools showing equivalent education. In lieu of such diplomas or certificates the applicant will be required to pass a matriculate examination which shall in each case be the equivalent of that forming the basis of the certificates of required preliminary education."

This places the Department of Dentistry on an equality with that of medicine in the same university in entrance requirements.

Time alone can determine the result of this high standard. While it undoubtedly will have a good effect in one direction, it may, on the other hand, if generally adopted, seriously interfere with the manipulative ability that has been a marked characteristic in the past of American dentistry.

